# Metabolomics and Proteomics Characterizing Hepatic Reactions to Dietary Linseed Oil in Duck

**DOI:** 10.3390/ijms232415690

**Published:** 2022-12-10

**Authors:** Yang Zhang, Ao Zhang, Laidi Wang, Ting Yang, Bingqiang Dong, Zhixiu Wang, Yulin Bi, Guohong Chen, Guobin Chang

**Affiliations:** Key Laboratory of Animal Genetics and Breeding and Molecular Design of Jiangsu Province, Yangzhou University, Yangzhou 225009, China

**Keywords:** PUFA, liver, duck, FADS2, glycerophospholipid

## Abstract

The imbalance in polyunsaturated fatty acid (PUFA) composition in human food is ubiquitous and closely related to obesity and cardiovascular diseases. The development of n-3 PUFA-enriched poultry products is of great significance for optimizing fatty acid composition. This study aimed to improve our understanding of the effects of dietary linseed oil on hepatic metabolism using untargeted metabolomics and 4D label-free proteome analysis. A total of 91 metabolites and 63 proteins showed differences in abundance in duck livers between the high linseed oil and control groups. Pathway analysis revealed that the biosynthesis of unsaturated fatty acids, linoleic acid, glycerophospholipid, and pyrimidine metabolisms were significantly enriched in ducks fed with linseed oil. Meanwhile, dietary linseed oil changed liver fatty acid composition, which was reflected in the increase in the abundance of downstream metabolites, such as α-linolenic acid (ALA; 18:3n-3) as a substrate, including n-3 PUFA and its related glycerophospholipids, and a decrease in downstream n-6 PUFA synthesis using linoleic acid (LA; 18:2n-6) as a substrate. Moreover, the anabolism of PUFA in duck livers showed substrate-dependent effects, and the expression of related proteins in the process of fatty acid anabolism, such as FADS2, LPIN2, and PLA2G4A, were significantly regulated by linseed oil. Collectively, our work highlights the ALA substrate dependence during n-3 PUFA synthesis in duck livers. The present study expands our knowledge of the process products of PUFA metabolism and provides some potential biomarkers for liver health.

## 1. Introduction

The importance of polyunsaturated fatty acids (PUFAs) in health and disease has gained increasing attention [1]. PUFAs are fatty acids that have two or more double bonds. PUFAs can be further classified based on their carbon chain length and the position of the first double bond on the methyl terminal [2]. Among these, n-3 and n-6 PUFAs are of great concern. Linolenic acid (LA) and α-LA (ALA) are essential fatty acids (EFAs) that cannot be synthesized by humans or other vertebrates. While important PUFAs, such as arachidonic acid (ARA; 20:4n-6), eicosapentaenoic acid (EPA; 20:6n-3), and cis-4,7,10,13,16,19-docosahexaenoic acid (DHA; 22:6n-3), can be directly taken up from the diet, they can also be endogenously converted from other PUFAs [3]. The liver plays a major role in this process, wherein dietary LA and ALA are metabolized into other PUFAs by desaturases, elongases, and peroxisomal β-oxidation [4]. The insufficient dietary intake of n-3 PUFAs and the concomitant excessive intake of n-6 PUFAs are associated with obesity and cardiovascular diseases. However, the influence of n-3 PUFA supplementation on the metabolic status and biosynthetic pathways in organisms is not well understood.

Duck consumption is common in Asian and European countries [5]. Compared to red meats, poultry have a lower fat content, with higher PUFA and lower TFA concentrations, and are considered part of the longevity diet [6]. Much effort has been focused on approaches for improving lipid profiles, with much emphasis on n-3 PUFA. The liver plays the main role in lipogenesis, providing lipids destined for use by all tissues [7], and largely determines the composition of fatty acids in edible meat. Duck (*Anas platyrhynchos*), one of the most important waterfowl, is an ideal model for PUFA synthesis and deposition studies. The liver is one of the most dynamic organs in birds and it performs a diverse array of functions, including the metabolism of fat, carbohydrates, proteins, vitamins, and minerals, the removal of waste products, and detoxification [8]. In contrast to mammals, in birds, more fat is synthesized in hepatic tissue and less in adipose tissue. In addition, the entire steatosis process of the waterfowl liver is reversible and causes no lasting cirrhosis or necrosis in the liver [9]. This suggests that waterfowl have a mechanism that protects their liver from harm caused by severe hepatic steatosis.

Linseed oil, an important source of dietary ALA for humans, is incorporated into foods to improve cardiovascular health [10]. In poultry, linseed-oil- or flaxseed-supplemented diets are also used to increase n-3 PUFA levels and decrease n-6: n-3 PUFA ratios in eggs and meat to produce foods enriched in n-3 PUFA, which has many positive health benefits [11,12,13]. Our previous study showed that dietary linseed oil can improve n-3 PUFA-enriched duck products [14]. However, changes in downstream lipid metabolites involved in the PUFA metabolic network affected by linseed oil in duck livers have not been investigated. In this study, Runzhou white-crested ducks, a popular duck breed with a small body size, were used to assess the effects of replacing duck oil with linseed oil on plasma biochemical parameters, liver microscopic structure, liver metabolomics, and the proteome. This study aims to provide a comprehensive understanding of n-3 PUFA synthesis, which is important for enriching n-3 PUFAs in poultry meat production. Furthermore, these insights into hepatic PUFA biosynthesis and metabolism in ducks may provide a reference for future human PUFA studies.

## 2. Results

### 2.1. Effects of Linseed Oil on Liver Morphology and Blood Parameters

**Figure 1:**
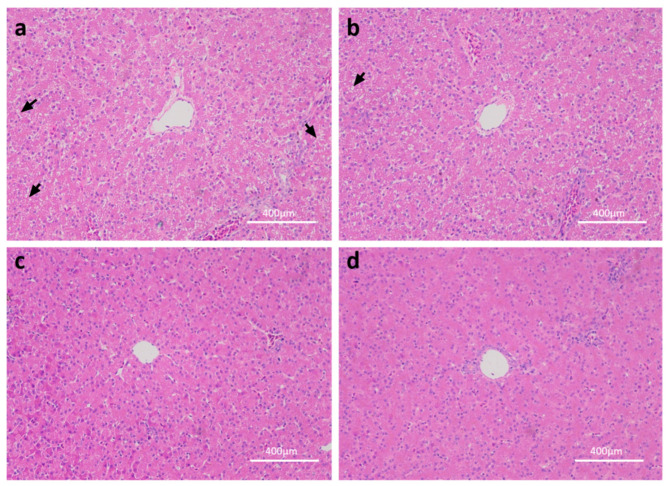
Representative images of stained hepatic sections with H&E. The arrow points to the typically gathered small white fat cavities: (**a**) is for the control group, (**b**) is for the low linseed oil group, (**c**) is for the medium linseed oil group, and (**d**) is for the high linseed oil group.

Morphometric and histological analyses (Figure 1) revealed that the central vein and hepatic nucleus were obviously observed, and the small white fat cavities were reduced as linseed oil increased. These analyses showed that dietary linseed oil reduced hepatic steatosis.

**Figure 2:**
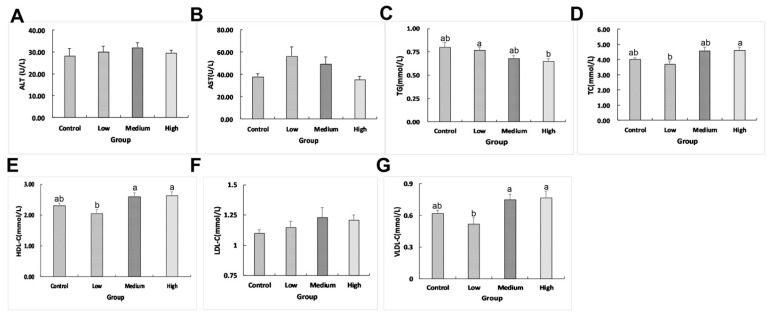
Effects of linseed oil on duck plasma parameters (*n* = 12). (**A**) Alanine aminotransferase (ALT) activities. (**B**) Aspartate aminotransferase (AST) activities. (**C**) Total triglyceride (TG) concentrations. (**D**) Total cholesterol (TC) concentrations. (**E**) High-density lipoprotein cholesterol (HDL-C) concentrations. (**F**) Low-density lipoprotein cholesterol (LDL-C) concentration. (**G**) Very-low-density lipoprotein cholesterol (VLDL-C) concentrations. ^a,b^ Values with different small letter superscripts mean statistical difference (*p* < 0.05). Low means low linseed oil group, medium means medium linseed oil group, high means high linseed oil group, and control means the control group. Error bar is drawn by the standard error of mean (SEM).

Plasma total triglyceride (TG), total cholesterol (TC), high-density lipoprotein cholesterol (HDL-C), and very-low-density lipoprotein cholesterol (VLDL-C) were significantly affected (*p_TG_* = 0.018 < 0.05, *p_TC_* = 0.014 < 0.05, *p_HDL-C_* = 0.006 < 0.05, *p_VLDL-C_* = 0.008 < 0.01) by the linseed oil diet. There were no significant changes (*p_ALT_* = 0.183 > 0.05, *p_AST_* = 0.094 > 0.05, *p_LDL-C_* = 0.190 > 0.05) in alanine aminotransferase (ALT) activities, aspartate aminotransferase (AST) activities, and low-density lipoprotein cholesterol (LDL-C) concentration between the linseed oil and control groups (Figure 2A–G). The ducks in the medium- and high-linseed oil group exhibited higher plasma HDL-C (*p_medium_ =* 0.031 < 0.05, *p_high_* = 0.012 < 0.05) and VLDL-C (*p_medium_ =* 0.040 < 0.05, *p_high_* = 0.021 < 0.05) concentrations than those in the low-linseed oil group. Plasma TG concentrations were lower in the high linseed oil group than that in the low linseed oil group (*p* = 0.047 < 0.05), while TC concentrations were higher in the high linseed oil group than in the low linseed oil group (*p* = 0.029 < 0.05).

### 2.2. Metabolomic Changes in Linseed-Oil-Supplemented Duck Livers

**Figure 3:**
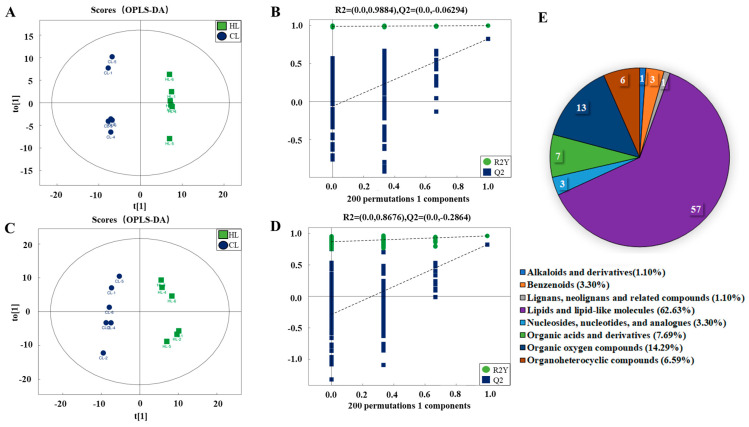
Effects of dietary linseed oil on hepatic metabolomic alterations (*n* = 6). OPLS−DA score plots in positive (**A**) and negative (**B**) ion modes for duck livers in high linseed oil group and the control group. Permutation test in positive (**C**) and negative (**D**) ion mode for duck livers in high linseed oil group and the control group. (**E**) Classification of significantly varying metabolites between the high linseed oil group and control group.

Eight samples were analyzed using ultra-high-performance liquid chromatography-mass spectrometry (UHPLC-MS). Orthogonal partial least squares-discriminant analysis (OPLS-DA) was employed to visualize the gas chromatography-mass spectrometer (GC-MS) dataset and display the similarities and differences among the samples. As shown in Figure 3A–D, there was a clear separation between the high linseed oil group and control group based on the OPLS-DA plot in the positive and negative ion modes, indicating that dietary linseed oil affected the metabolite components in duck livers.

**Figure 4:**
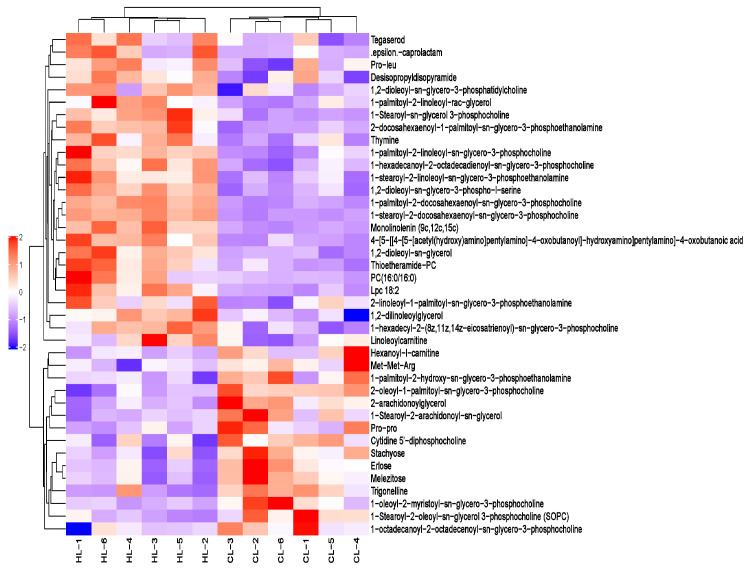
Heat map of differential metabolites in the positive ion modes. Each line means a metabolite and each column a sample; HL1–HL6 represent replicates in the high linseed oil group and CL1–CL6 in the control group. The upregulated metabolites are shown in red color, whereas the downregulated metabolites are presented in blue color.

**Figure 5:**
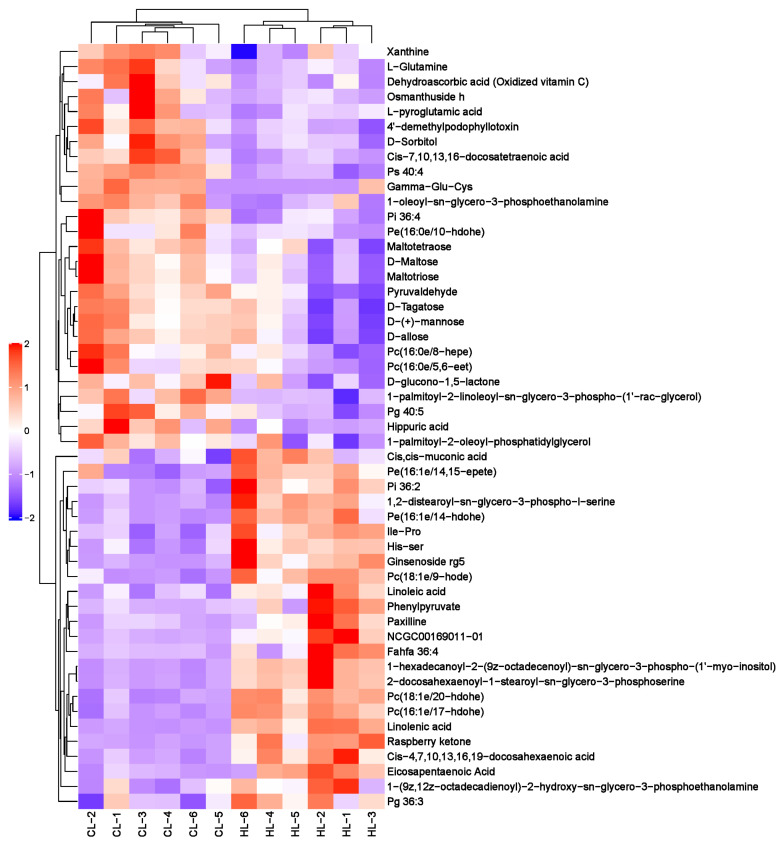
Heat map of differential metabolites in the negative ion modes. Each line means a metabolite and each column a sample; HL1–HL6 represent replicates in the high linseed oil group and CL1–CL6 in the control group. The upregulated metabolites are shown in red color, whereas the downregulated metabolites are presented in blue color.

A total of 1999 liver metabolites were identified, of which 1255 were identified in positive ion mode and 744 in negative ion mode. Based on the OPLS-DA VIP > 1 and *p* value < 0.05, 91 differential metabolites were ultimately obtained, including 25 for upregulation and 15 for downregulation in positive ion mode and 24 for upregulation and 27 for downregulation in negative ion mode (Supplementary Tables S1 and S2). Overall, the differential metabolites mainly belonged to lipids and lipid-like molecules (62.63%), organic oxygen compounds (14.29%), organic acids and derivatives (7.69%), and organoheterocyclic compounds (6.59%) (Figure 3E). These results indicated that linseed oil caused changes in lipid metabolism and oxidation pathways in duck livers. The heat map analysis for differential metabolite levels in every sample between the groups is shown in Figure 4 and Figure 5.

As for lipids and lipid-like molecules, fatty acids such as ALA, EPA, DHA, and LA, were upregulated, while Cis-7,10,13,16-docosatetraenoic acid (DTA) was downregulated. The downstream fatty acids acyl, monolinolenin (MLN), 1,2-dilinoleoylglycerol (DLG), 1,2-dioleoyl-sn-glycerol (DOG), and 1-palmitoyl-2-linoleoyl-rac-glycerol (PLG) were upregulated, whereas 2-arachidonoylglycerol (2-AG) and 1-stearoyl-2-arachidonoyl-sn -glycerol (SAG) were downregulated. Dietary linseed oil decreased the expression of hexanoyl-l-carnitine (HLC) and increased the expression of linoleoylcarnitine (LLC). There were 36 identified types of glycerol phospholipids. Downstream glycerophospholipids ALA and LA, 1-palmitoyl-2-linoleoyl-sn-glycero-3-phosphocholine (PLGPC), 1,2-dioleoyl-sn-glycero-3-phosphatidylcholine (DOGPC), lyso-phosphatidylcholine (LPC; 18:2), 1-stearoyl-2-linoleoyl-sn-glycero-3-phosphoethanolamine (SLGPE), and 2-linoleoyl-1-palmitoyl-sn-glycero-3-phosphoethanolamine (LPGPE) were upregulated. The downstream glycerophospholipids of DHA, 1-stearoyl-2-docosahexaenoyl-sn-glycero-3-phosphocholine (SDGPC), 1-palmitoyl-2-docosahexaenoyl-sn-glycero-3-phosphocholine (PDGPC), and 2-docosahexaenoyl-1-palmitoyl-sn-glycero-3-phospho-ethanolamine (DPGPE) were upregulated. In contrast, 1-stearoyl-2-oleoyl-sn-glycerol-3-phosphocholine (SOGPC), 1-oleoyl-2-myristoyl-sn-glycero-3-phosphocholine (OMGPC), 2-oleoyl-1-palmitoyl-sn-glycero-3-phosphocholine (OPGPC), and 1-octadecanoyl-2-octadecenoyl-sn-glycero-3-phosphocholine (OOGPC) were downregulated. Generally, linseed oil caused changes in glycerophospholipids formed by 16 C and 18 C compounds and DHA.

Most of the organic oxygen compounds, Melezitose, Erlose, Stachyose, Maltotriose, D-allose, D-Maltose, D-Sorbitol, Maltotetraose, D-(+)-mannose, D-Tagatose, D-glucono-1,5-lactone, and Osmanthuside h Pyruvaldehyde, were downregulated. As for organic acids and derivatives, desisopropyldisopyramide and Pro-leu were upregulated, whereas Met-Met-Arg, Pro-pro, Gamma-Glu-Cys, L-glutamine, and L-pyroglutamic acid were downregulated. In addition, raspberry ketone, paxilline, phenylpyruvate, His-ser, and epsilon-caprolactam were upregulated.

To further evaluate the molecular function of these differential metabolites in duck livers, all differential components in each group were mapped to the Kyoto Encyclopedia of Genes and Genomes (KEGG) database. The top 20 KEGG pathways were enriched in the control and high linseed oil groups (Supplementary Figure S1). Metabolic pathways involving 23 differential metabolites were the most enriched in the pathway enrichment analysis. The biosynthesis of unsaturated fatty acids, linoleic acid metabolism, and ATP-binding cassette (ABC) transporters were enriched. There was a focus on the pathways with more than four differential metabolites. Of the five differential metabolites involved in the biosynthesis of unsaturated fatty acid pathways, LA, ALA, EPA, and DHA were upregulated and DTA was downregulated. In addition, 23 different metabolites were involved in the metabolic pathway, which covered a wide range of metabolic pathways, indicating that ALA caused changes in liver material metabolism. Eleven metabolites were downregulated and one was upregulated in the ABC transporter pathway. The retrograde endocannabinoid signaling pathway mainly involves four different metabolites, among which 2-AG, SOGPC, and SAG were downregulated and phosphatidylcholine (PC) (16:0/16:0) was upregulated.

### 2.3. Proteomics Changes in Linseed-Oil-Supplemented Duck Livers

**Figure 6:**
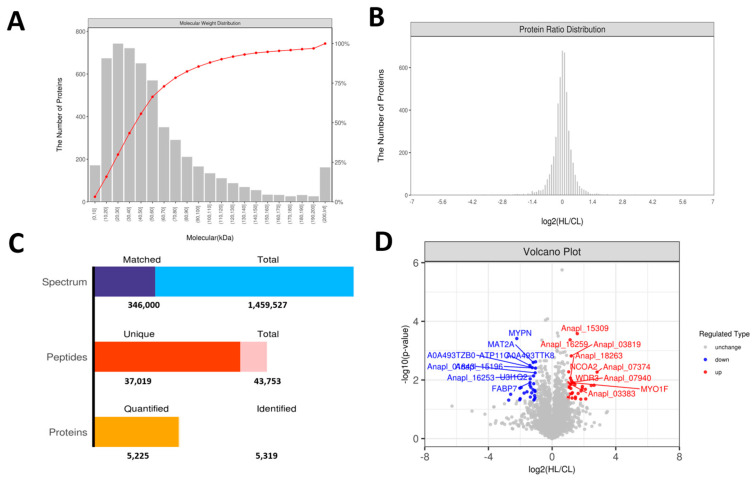
Effects of dietary linseed oil on hepatic proteomic alterations (*n* = 4). (**A**) Identification peptide number distribution. The abscissa is the relative molecular weight of the identified proteins. The left ordinate represents the number of proteins; the subordinate represents the cumulative percentage of proteins with a relative molecular mass no higher than that of the corresponding. (**B**) Protein abundance ratio map. (**C**) Statistical diagram of identification and quantitative results. Total spectrum: total number of secondary spectra, matched spectrum: total number of matched spectra, total peptide: total number of unique peptides, identified proteins: quantified proteins can be quantified. (**D**) Volcano diagram of differentially expressed proteins.

**Figure 7:**
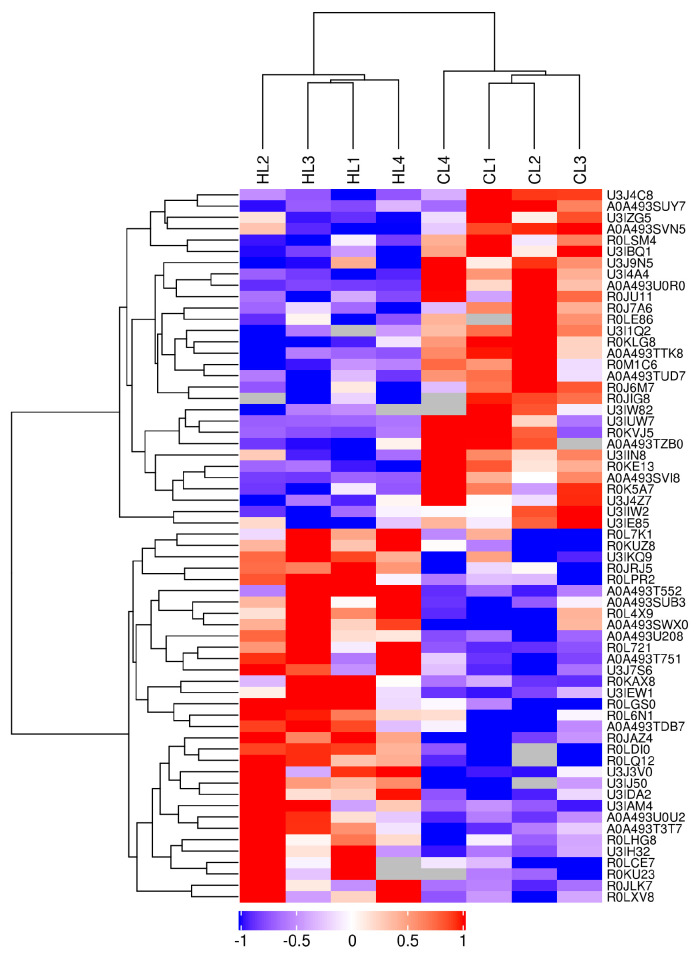
Cluster heat map of differentially expressed proteins.

Identification and quantification of proteins and peptides were carried out based on the mass spectrometry results, as shown in Figure 6A,B. In total, 1,459,527 spectrograms were obtained, with 43,753 identified peptides, 5319 identified proteins, and 5225 quantifiable proteins (Figure 6C). According to the criteria of |log2 (FoldChange)| > 1 and *p* < 0.05, 63 proteins exhibited significant differential expression between the two groups (Figure 6D). Of these, 33 proteins were upregulated and 30 were downregulated in the high linseed oil group compared to those in the control group (Figure 7 and Supplementary Table S3).

The Gene Ontology (GO) annotation statistics of the differentially expressed proteins are shown in Supplementary Figure S2. Cellular process and metabolic process (BP), binding and catalytic activity (MF), and cell and cell part (CC) were the main terms involved in the differential expression of proteins. The top 20 enriched pathways by differentially expressed proteins are shown in Supplementary Figure S3. Notably, 63 proteins were found to be associated with ALA metabolic pathways, glyceryl ester and glycerol phospholipid metabolism pathways, the polyunsaturated fatty acid synthesis pathway, the TCA (tricarboxylic acid) cycle pathway, and primary bile acid biosynthesis. The protein level of fatty acid desaturase 2 (FADS2) In the synthesis process of polyunsaturated fatty acids decreased, and the expression of lipin 2 (LPIN2) and ETNNPL in the metabolism pathway of ALA was mainly upregulated. Phospholipase A2-IVA (PLA2G4A) was downregulated in the glyceryl ester and glycerol phospholipid metabolism pathways. In addition, these pathways are involved in PRKAA2, Myosin 1F (MYO1F), fatty acid-binding protein 7 (FABP7), myopalladin (MYPN), and kinesin family member 21A (KIF21A).

### 2.4. Conjoint Analysis of Differential Metabolites and Proteins

**Figure 8:**
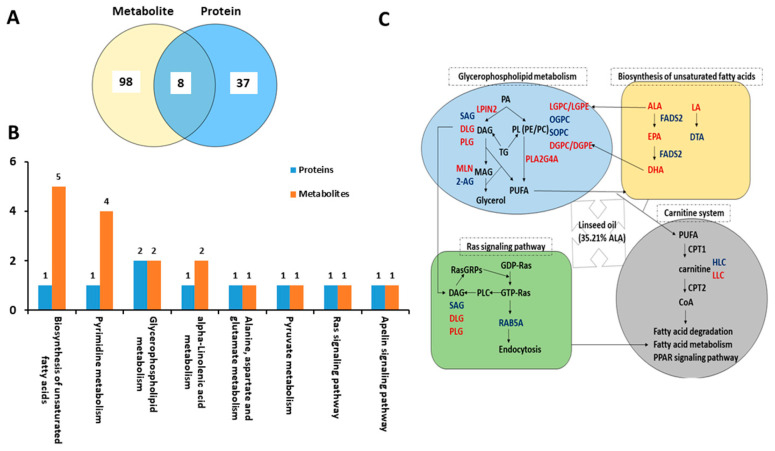
Combined effects of linseed oil on the metabolome and proteome of duck livers. (**A**) Venn diagram of the enriched pathways. (**B**) Bar graph analysis of the common pathways. (**C**) The pathway diagram of key metabolites and proteins in the liver caused by dietary linseed oil.

**Figure 9:**
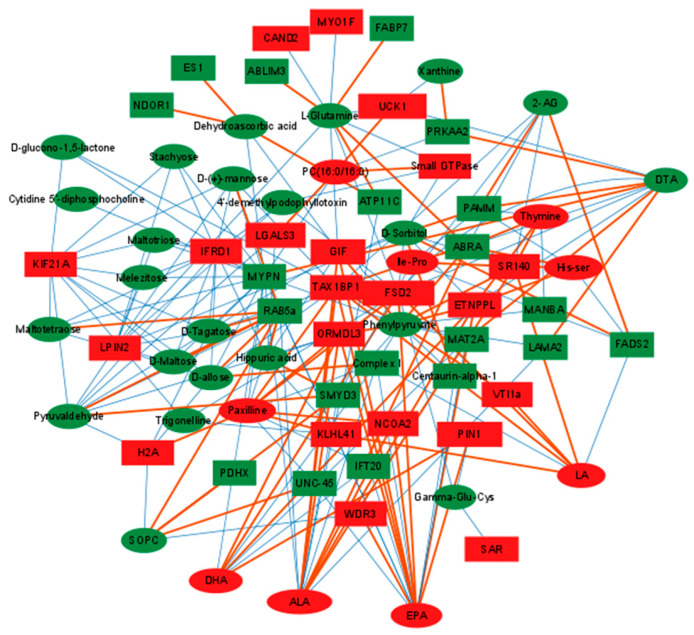
Differential metabolite–protein interaction networks. Circle means differential metabolites, rectangle means differential proteins; red represents upregulated, green represents downregulated; orange line represents positively related, blue represents negatively related.

Eight common signaling pathways were enriched by both differential proteins and metabolites, including the biosynthesis of unsaturated fatty acids, glycerophospholipid metabolism, and ALA metabolism pathways (Figure 8A,B). Important altered pathways in the duck liver affected by dietary linseed oil are shown in Figure 8C. Interestingly, three proteins and related metabolites were mapped to these pathways, FADS2, LPIN2, and PLA2G4A, and several fatty acids (LA, ALA, EPA, DHA, and DTA). PUFA-FADS2-PUFA, PA-LPIN2-DAG, and PL-PLA2G4A-PUFA played an important role in ALA-induced metabolic changes in the liver (Figure 8C). To investigate the relationship between metabolic and proteomic variations in duck livers affected by dietary linseed oil, 91 differential metabolites and 63 differential proteins were identified and subjected to Pearson correlation hierarchical clustering analysis. Related metabolites and proteins in the same samples according to the association between the protein and the metabolite in the sample, based on |r| ≥ 0.5 and *p* < 0.05, are shown in Supplementary Figure S4. The interaction network of molecules that may play important roles in lipid metabolism in the liver was constructed (Figure 9). The level of 2-AG was positively related to FADS2 and negatively related to the Tax1-binding protein 1-like protein, and the level of n-3 PUFA (ALA, EPA, and DHA) was positively related to ORM1-like protein 3 and negatively related to MYPN.

## 3. Discussion

### 3.1. Linseed Oil Improved Blood Parameters

The liver is closely associated with blood lipid metabolism. While the main products of de novo hepatic lipogenesis are triglycerides, the liver is also the major site of phospholipid and cholesterol synthesis. These lipids, along with proteins, are components of lipoproteins. In birds, apart from portomicrons, which transport lipids from the gastrointestinal tract to the liver via portal circulation, lipoproteins (very-low-density lipoprotein, VLDL; low-density lipoprotein, LDL; high-density lipoprotein, HDL) are synthesized in the liver and released into the bloodstream, transporting lipids to other tissues [15]. HDL-C is formed from HDL and free cholesterol in the blood. It can enter liver cells for metabolism by the SR-B1 receptor and is eventually excreted from the bile [16]. Therefore, blood HDL-C levels reflect the reversal of cholesterol [17]. In the present study, we found that dietary linseed oil ameliorated liver conditions and reduced fat deposition. This coincides with results from a study of largemouth bass (*Micropterus salmoides)* [18], which indicated that linseed oil can improve liver function and antioxidant ability of largemouth bass. Studies have reported that long-chain n-3 PUFA supplementation reduces serum TG concentrations [11,19] and that ALA-rich vegetable oils lower serum TG levels in rats [20]. Our findings also showed that ducks fed high levels of linseed oil had lower plasma TG concentrations than the control and low linseed oil groups. In contrast, blood plasma TC and HDL concentrations were higher in the high linseed oil group than in the control and low linseed oil groups, consistent with previous reports [21,22]. There was no significant difference in these plasma biochemical indices between the medium and high linseed oil groups. Therefore, it can be concluded that the increase in HDL-C after the addition of linseed oil promoted the decomposition of plasma TG and there was no significant difference in the effect of 1% and 2% linseed oil on blood lipids.

### 3.2. Dietary Linseed Oil Altered the Lipid Composition of the Liver

Metabolites are the final products of all cellular activities and are sensitive markers of physiological activity. In the present study, most n-3 PUFAs were upregulated, including ALA, EPA, and DHA. Notably, our findings are consistent with those of previous studies [13,23,24,25,26]. As for n-6 PUFA, LA was upregulated and DTA was downregulated. Our results also showed that the addition of linseed oil caused an increase in downstream acyl glycerols, such as MLN, DLG, and PLG, with ALA and LA as substrates. It has been reported that the addition of monoacylglycerols, containing a saturated acyl chain from 12 to 20 carbons, slowed plasma clearance and decreased liver uptake of the remnants, while monoacylglycerols with unsaturated acyl chains, such as monoarachidonin (M20:4), monolinolenin (M18:3), monolinolein (M18:2), and monoolein, could mitigate these effects [27]. Other studies have also demonstrated adverse effects of saturated acyl chains on the residual clearance of chylomicrons in rats [28]. The increase in unsaturated acyl glycerol levels caused by ALA metabolism after the addition of linseed oil may play a regulatory role in the clearance of porous particles in poultry. In addition, HLC and LLC, downstream products of LA oxidative utilization, were upregulated. This indicates that LA enters the mitochondria through the fatty acylcarnitine system and oxidative utilization is increased.

A metabolomic study found that ARA levels downstream of acyl glycerol (2-AG and SAG) in the liver of the high linseed oil group significantly decreased. There was a downward trend in the levels of arachidonic acid-derived endocannabinoid anandamide (AEA). This is consistent with the results of Ulrike et al. (2016) [29], who compared the effects of pork and cod on mice fed a Western diet and found that the levels of AEA and 2-AG in the liver of the cod group decreased. Human studies have also shown that overweight and obese individuals often have higher circulating levels of 2-AG and AEA and an altered pattern of receptor expression, which affects the endocannabinoid signaling system [30]. Consequently, this leads to an increase in orexigenic stimuli and changes in fatty acid synthesis, insulin sensitivity, and glucose utilization, with preferential energy storage in adipose tissue. As endocannabinoids are products of dietary fats, modification of dietary intake may modulate their levels, with EPA- and DHA-based endocannabinoids being able to displace ARA from cell membranes, thereby reducing AEA and 2-AG production [30]. This alleviates the development of obesity and reduces the accumulation of liver lipids.

### 3.3. Dietary Linseed Oil Altered the Level of Hepatic Lipid-like Molecules

Hepatic lipogenesis occurs through a series of chain reactions, including glycolysis, the citric acid cycle, and fatty acid synthesis. PUFA can be combined with triglycerides (TG and TAG) to produce phosphatidic acid (PA) and phospholipids (PL) in vivo. We found that the downstream glycerophospholipids of LA, ALA, and DHA (PLGPC, SLGPE, LPGPE, SDGPC, PDGPC, and DPGPE) were upregulated, whereas most glycerophospholipids involved in oleoyl (SOGPC, OMGPC, and OPGPC) were downregulated. It has been reported that dietary n-3 PUFAs are preferentially incorporated into PL compared to triglycerides. Phospholipids are essential components of all cellular and subcellular membranes, with PC and phosphatidylethanolamine (PE) being the most abundant [31], and they can form lipid bilayers. Feed-derived n-3 PUFAs can be converted into phospholipids of biofilms, which can enhance the function of transmembrane proteins and their interaction with extracellular ligands, thereby affecting membrane fluidity [32,33]. This also indirectly affects cell membrane signaling pathways and other physiological functions.

Lyso-phosphatidylcholine (LPC), the product of enzymatic hydrolysis of PC, is an important lipid mediator involved in cell metabolism. Direct hepatic secretion is an important source of large amounts of unsaturated LPC in plasma [34]. Palmitoyl-, oleoyl-, linoleyl-, and arachinyl-LPC 16:0, 18:1, 18:2, and 20:4 are the most important types of LPC produced by endothelial lipase (EL). Low plasma LPC 18:2, which has previously been shown to predict impaired glucose tolerance, insulin resistance, type 2 diabetes, coronary artery disease, and memory impairment, is an independent predictor of gait speed decline in older adults [35]. Our results indicate that dietary ALA supplementation resulted in the upregulation of LPC 18:2 in the liver. However, the important mechanisms of specific LPC fatty acyl chains in metabolic regulation remain unclear. Past studies have demonstrated the role of saturated or monounsaturated LPC in promoting inflammation and atherosclerosis [36,37,38], and the esterification of n-3 PUFAs to ethyl esters or TGs is beneficial to human health [39].

### 3.4. Dietary Linseed Oil Altered the Metabolism of PUFA in Liver

Overall, dietary linseed oil caused the downstream fatty acid esters or glycerophospholipids of LA, ALA, and DHA to be upregulated, while the fatty acid esters of ARA and the glycerophospholipids involved in oleoyl were downregulated. Downregulation of FADS2 protein levels did not affect the increase in n-3 PUFA (EPA and DHA) synthesis, suggesting that the anabolism of PUFA in duck livers has obvious substrate-dependent effects. This is consistent with the results of previous studies [14,25]. Furthermore, the increase in ALA, LA, and DHA led to an increase in the levels of the corresponding downstream lipoacyl-related metabolites, while the levels of the downstream oleacyl-related metabolites decreased. This is consistent with the findings of Singer et al. (1990) [40] regarding the effects of fish oil supplementation on liver metabolites in hypertensive rats, who found that feeding fish oil led to a significant increase in EPA and DHA in liver TG, PC, and PE at the expense of n-6 PUFA (except LA in PC and PE). This can provide an important reference for human n-3 PUFA diet research. Studies have also shown that feeding n-3 PUFA or n-3 PUFA phosphatidylcholine-enriched lipids alleviated hepatic lipid accumulation by suppressing lipogenic gene expression in the liver [41].

Typical n-3 PUFAs (DHA and EPA) are competitive substrates for enzymes and products of ARA metabolism. DHA- and EPA-derived eicosanoids antagonize the pro-inflammatory effects of n-6 fatty acids; n-3 PUFAs downregulate inflammatory genes and lipid synthesis and stimulate fatty acid degradation. In addition, the n-3/n-6 PUFA content of cell and organelle membranes, as well as membrane microdomains, strongly influences membrane function and numerous cellular processes, such as cell death and survival [42]. In addition, bioactive lipids are generated via the hydrolysis of membrane lipids mainly by phospholipases, giving rise to fatty acids and lysophospholipids that either directly exert their function or are further converted to active mediators [36]. The levels of ARA, EPA, and DHA in PUFAs released from PC metabolism may have a considerable impact on the immune system function. Oxidation of ARA produces pro-inflammatory two-series prostaglandins (PGE2), leukotrienes (LTB2), and thromboxanes (TBX2) [38]. Eicosapentaenoic acid can increase the anti-inflammatory three-series PG, PGI, and TX [37]. Docosahexaenoic acid is metabolized into anti-inflammatory resolvins and neuroprotectins [43]. These lipid mediators inhibit the generation of two series of prostaglandins, leukotrienes, and thromboxanes. These metabolites were not detected in this study, which may be because the amount generated was too low.

Past studies on the effects of linseed oil on the body mainly focused on the gene transcription level but less on the protein level [14,22,24]. The synthesis of n-3 PUFA increased under the catalytic action of FADS2, which competitively inhibited the synthesis of n-6 PUFA using LA as a substrate due to the increase in ALA substrate in the liver after linseed oil supplementation. This is consistent with the results of a previous study [22,26]. LPIN2 is a member of the lipin family, which acts as a phosphatidate phosphatase enzyme and plays an important role in the de novo biosynthesis of triacylglycerol, phosphatidylcholine, and phosphatidylethanolamine [44]. Our study further demonstrated that linseed oil can regulate the pathway of phosphatidic acid (1,2-dilipoacyl-sn-glycerol-3-phosphate) hydrolysis in the liver to diglycerol in response to the increased expression of LPIN2. PLA2G4A, the most abundant subtype of cytosolic phospholipase A2, mediates the release of PUFAs such as ARA, EPA, or DHA from phosphatidylcholine PC, thereby affecting prostaglandin production and function [45]. The results showed that different proteins were associated with different metabolites, which jointly affected the fatty acid composition and metabolism of the liver.

Differentially expressed proteins are also involved in muscle development, differentiation, and muscle components. The differentially expressed proteins mainly included downregulated proteins MYPN, calsequestrin 2 (CASQ2), and SMYD3, and upregulated proteins KLHL41 and MYO1F. Few studies have investigated the effects of dietary linseed oil on the expression of these genes. However, studies have shown that DHA can improve muscle homeostasis by boosting muscle protein synthesis or slowing muscle protein degradation [46]. The n-3 PUFAs have been shown to reduce the occurrence of sarcopenia in the elderly by positively modulating intracellular metabolic signaling [47]. MYPN encodes Z-line proteins related to sarcomeric integrity. MYPN-knockout mice showed a 48% reduction in muscle fiber cross-sectional area and a significant increase in fiber number [48]. Kelch-like protein 41 (KLHL41) is polyubiquitinated and mutant mice exhibit lethal sarcomere destruction and abnormal expression of muscle structure and contractile proteins [49]. Myosin IF (MYO1F) can act by regulating the acetylation of α-tubulin and microtubules [50]. Methyltransferase SMYD3 can mediate the transcriptional cofactor recruitment of myostatin and c-Met genes and regulate skeletal muscle atrophy. Inhibition of SMYD3 can prevent muscle loss and fiber size reduction [51] and has been shown to promote the proliferation and metastasis of liver cancer [52]. These genes are related to muscle function; however, the specific mechanism of these differentially expressed proteins in the liver after linseed oil addition is not clear.

## 4. Materials and Methods

### 4.1. Animals and Experimental Design

All animal-related work was approved by the Yangzhou University Animal Care and Use Committee (2020-0131). All protocols and procedures were performed according to the Chinese Regulations for Laboratory Animals.

A total of 224 28-day-old Runzhou white-crested ducks from the commodity generation (average weight: 753.35 ± 12.94 g) were randomly divided into four treatment groups. Each treatment group had four replicates, each consisting of 14 ducks (half male and half female). The four treatment groups were fed basic diets (Supplemental Table S4) supplemented with control (2% duck oil), low linseed oil (1.5% duck oil + 0.5% linseed oil), medium linseed oil (1% duck oil + 1% linseed oil), and high linseed oil (2% linseed oil). Duck oil is a product which is boiled and then filtered from waste duck abdominal fat. Linseed oil is obtained from linseed by physical pressing. The main difference in fatty acid composition between linseed oil and duck oil is the ALA content. The ALA content of linseed oil in this experiment was 35.21%, that of duck oil was 0.67% (Supplementary Table S5), and the content of mixed oil (linseed oil and duck oil) added to the basic diet was 2%. According to the amount of the basic diet, the corresponding proportion of oil was accurately weighed, then a small amount was premixed and then all mixed until the oil was evenly distributed in the pellet feed. The dietary ALA content increased from 0.08% to 0.68% with increasing linseed oil ratio (Supplementary Table S5). The birds were raised on a floor system laid by deep litter comprising sawdust and paddy husk. All ducks had ad libitum access to food and water. The feeding experiment lasted for 28 d and was conducted at Zhenjiang Tiancheng Agricultural Technology Co. Ltd., Zhenjiang, Jiangsu Province, China.

### 4.2. Blood and Sample Collection

Following a 12 h fast, six ducks (three males and three females) from each replicate were selected, and the blood samples were collected via wing vein puncture, then plasma was obtained after centrifugation of blood samples at 3500× *g* for 10 min at 4 °C and stored at −20 °C. After bleeding, birds were sacrificed by stunning, and three small portions of the right middle liver were collected, immediately placed in liquid N2, and stored at −80 °C for metabolomic and proteomic analysis. In addition, 1 × 1 × 1 cm samples were excised from each bird from the middle part of the right liver lobe and immersed in neutral buffered formalin (4%, pH 7, 20–24 °C) for subsequent histological analysis.

### 4.3. Plasma Biochemical Parameters

Plasma aspartate aminotransferase (AST), alanine aminotransferase (ALT), total cholesterol (TC), total triglyceride (TG), high-density lipoprotein cholesterol (HDL-C), and low-density lipoprotein cholesterol (LDL-C) concentrations were determined using Roche-Hitachi Cobas 8000 (Roche, Indianapolis, IN, USA) and matched commercial kits (Roche, Munich, Germany). The very-low-density lipoprotein cholesterol (VLDL-C) concentration was calculated as follows:

C_TC_ = C_HDL-C_ + C_LDL-C_ + C_VLDL-C_.

### 4.4. Liver Morphological Observation

Liver samples were dehydrated in an ascending graded series of ethanol, embedded in paraffin, serially sectioned at 5 μm, and stained with Meyer’s hematoxylin and eosin (H&E). All procedures were performed by Wuhan Servicebio Technology Co., Ltd. (Wuhan, China). The stained sections were observed using a Leica Autostainer XL (Leica Biosystems, Wetzlar, Germany).

### 4.5. Untargeted Metabolomic Analysis of Liver

After thawing slowly at 4 °C, liver samples were added to a precooled methanol/acetonitrile/water solution (2:2:1, *v*/*v*) for metabolite extraction. The mixture was centrifuged at 12,000× *g* at 4 °C for 20 min. The supernatant was dried using a vacuum centrifuge. For LC-MS analysis, the samples were re-dissolved in 100 μL acetonitrile/water (1:1, *v*/*v*) solvent and filtered through a 0.22 μm membrane. Detection was performed by Shanghai Applied Protein Technology Co., Ltd, Shanghai, China.

The samples were subjected to an Agilent 1290 Infinity LC ultra-high-performance liquid chromatography UHPLC system (Agilent, Sant Clara, CA, USA) coupled with an AB Triple TOF 6600 mass spectrometer (SCIEX, Washington, DC, USA) to analyze the metabolic profiles in both electrospray ionization (ESI) positive and negative ion modes. Chromatographic separation was carried out on an ACQUITY UPLC BEH Amide column (100 mm × 2.1 mm, 1.7 μm, Waters Corporation, Milford, MA, USA) equipped with a binary solvent system (solvent A: water + 25 mM ammonium acetate + 25 mM ammonia water; solvent B: acetonitrile). The gradient was 85% for 1 min, linearly decreased to 65% over 11 min, then to 40% over 0.1 min, and was then maintained for 4 min. Then, it was increased to 85% in 0.1 min and a 5 min re-equilibration period was employed. The gradients were at a flow rate of 0.4 mL/min, the injection volume was 2 μL, and the column temperature was maintained at 25 °C.

The ESI source conditions were set as follows: Ion Source Gas1 of 60, Ion Source Gas2 of 60, curtain gas of 30, source temperature of 600 °C, and IonSpray Voltage floating of ±5500 V. In MS acquisition, the instrument was set to acquire over the *m*/*z* range of 60–1000 Da and the accumulation time for the TOF MS scan was 0.20 s/spectra. In the auto MS/MS acquisition, the instrument was set to acquire over the m/z range of 25–1000 Da and the accumulation time for the product ion scan was 0.05 s/spectra. The product ion scan was acquired using information-dependent acquisition with a high-sensitivity mode. The parameters were set as follows: collision energy (CE) fixed at 35 V with ±15 eV; declustering potential (DP), 60 V (+), and −60 V (−), excluding isotopes within 4 Da; and 10 candidate ions to monitor per cycle.

Raw data were converted into mzXML files using Proteowizard software (v3.0.8789). The XCMS package in R (v3.3.2) was used for peak identification, peak filtration, and peak alignment. Peak area data were normalized within the sample. Collection of Algorithms of MEtabolite pRofile Annotation (CAMERA) was used for the annotation of isotopes and adducts. Compound identification of metabolites was performed by comparing the accuracy of the *m/z* value (<10 ppm) and MS/MS spectra with an in-house database established with available authentic standards. After sum normalization, the processed data were analyzed using the R package (ropls), including two multivariate data analysis methods: pareto-scaled principal component analysis (PCA) and orthogonal partial least squares-discriminant analysis (OPLS-DA). The variable importance in the projection (VIP) value of each variable in the OPLS-DA model was calculated to indicate its contribution to classification. Student’s *t*-test was used to compare the differences between the two groups. VIP > 1 and *p* value < 0.05 were used to screen significantly changed metabolites. Pearson’s correlation analysis was performed to determine correlations between the two variables.

### 4.6. The 4D Label-Free Proteome Analysis of Liver

Eight duck liver samples from the high linseed oil and control groups were chosen for label-free proteome analysis. Approximately 20 mg of liver sample was lysed in SDT (4% SDS, 100 mM Tris-HCl, 1 mM DTT, pH 7.6) for protein extraction. The protein concentration was determined using a bicinchoninic acid assay kit (Pierce Chemical Company, Rockford, IL, USA). Approximately 20 µg of protein was added to the 5× loading buffer and bathed in boiling water for 5 min. Then, 12.5% SDS-PAGE electrophoresis (constant flow 14 mA, 90 min) was performed and visualized by Coomassie Blue R-250 staining. Protein was digested with trypsin according to the filter-aided sample preparation procedure described by Wiśniewski et al. (2009) [53]. The digested peptides of each sample were desalted on C18 Cartridges (Empore SPE Cartridges C18 (standard density), bed I.D. 7 mm, volume 3 mL, Sigma), concentrated by vacuum centrifugation, and reconstituted in 40 µL of 0.1% (*v*/*v*) formic acid.

LC-MS/MS analysis was performed on a timsTOF Pro mass spectrometer (Bruker) coupled to a nanoelute (Bruker Daltonics, Billerica, MA, USA) for 60/120/240 min. The peptides were loaded onto a reverse-phase trap column (Thermo Scientific Acclaim PepMap100, 100 μm × 2 cm, nanoViper C18, Waltham, MA, USA) connected to a C18 reversed-phase analytical column (Thermo Scientific Easy Column, 10 cm long, 75 μm inner diameter, 3 μm resin) in buffer A (0.1% formic acid) and separated with a linear gradient of buffer B (84% acetonitrile and 0.1% formic acid) at a flow rate of 300 nL/min controlled by IntelliFlow technology. The mass spectrometer was operated in the positive ion mode and collected ion mobility MS spectra over a mass range of 100–1700 m/z according to the manufacturer’s instructions.

Data were processed using MaxQuant software (v.1.5.3.17) against the Anas platyrhynchos UniProt proteome database (https://www.uniprot.org/taxonomy/8839) accessed on 20 August 2021. The parameters are listed in the supplementary data. Student’s *t*-test was used to evaluate significant differences. Proteins with a fold change of 1.20 or 0.83 and *p* value < 0.05 were considered differentially expressed. Cluster 3.0 (http://bonsai.hgc.jp/~mdehoon/software/cluster/software.htm) and Java Treeview software (http://jtreeview.sourceforge.net) both accessed on 10 November 2021 were used to perform hierarchical clustering analysis. CELLO (http://cello.life.nctu.edu.tw/), a multiclass SVM classification system, accessed on 10 November 2021 was used to predict subcellular protein localization. Differentially expressed proteins were locally searched for using the NCBI database (https://www.ncbi.nlm.nih.gov/protein/) accessed on 10 November 2021. GO terms were mapped and annotated using Blast2GO software. KEGG pathway enrichment analysis was performed to examine biological pathways (http://geneontology.org/) accessed on 8 May 2022. The results were visualized using Cytoscape software (version 3.8.2). Enrichment analysis was applied based on Fisher’s exact test, considering all quantified proteins as the background dataset.

### 4.7. Association Analyses of Differentially Expressed Metabolites and Proteins

PCA was performed using SIMCA (v.14.1) using quantitative data from the two omics. All differentially expressed proteins and metabolites were queried and mapped to pathways based on online KEGG (http://www.kegg.jp/) accessed on 8 May 2022. Enrichment analysis was also performed. R (v.3.5.1) was used to combine the KEGG annotation and enrichment results of the two omics methods. Venn diagrams and bar plots were constructed. Differentially abundant proteins and metabolites were Z-score-scaled (label-free) and concatenated into one matrix. The results of normal distribution test and homogeneity test of variance are shown in Supplementary Sheets S1 (proteins) and S2 (metabolites). Most of the data were in line with normal distribution and homogeneity of variance, so correlation coefficients among all molecules in the matrix were calculated using the Pearson algorithm in R (v.3.5.1). Pearson correlation coefficient among the differentially expressed proteins and metabolites were loaded into CytoScape (v.3.9.1) [54] and the correlation network was calculated.

### 4.8. Statistical Analysis

Data were analyzed using SPSS22.0 in a completely randomized design with a model containing treatment as the main effect. All data were checked for normality and homogeneity of variance before being analyzed (Supplementary Sheet S3). If the data were not normally distributed or the variance was not uniform, Kruskal–Wallis H tests were used for analysis. In addition, the Bonferroni method was used to correct the post hoc pairwise comparison of significance levels. If the data conform to normal distribution and the variance is homogenous, one-way ANOVA and the Duncan method were used for analysis. Results are expressed as the mean ± standard error of mean (SEM). Significance was set as *p* < 0.05. The experimental design and workflow are illustrated in Supplementary Figure S5.

## 5. Conclusions

The upregulated metabolites (MLN, DLG, LGPL, and DGPL) and downregulated metabolites (2-AG, SAG, and SOPC) may be the key marker metabolites in the liver caused by dietary linseed oil addition, and FADS2, LPIN2, and PLA2G4A may play important roles in PUFA metabolism. In addition, the plasma HDL-C level was increased by dietary linseed oil, which promoted triglyceride utilization and reduced fat accumulation in the liver. Therefore, research on hepatic PUFA metabolism in ducks provides references for n-3 PUFA-rich livestock and poultry production and the effects of n-3 PUFA on human health. Our results support the hypothesis that the duck liver plays a major role in n-3 PUFA levels, both from the diet and from endogenous conversion.

## Data Availability

Data are contained within the article.

## Supplementary Materials

The following supporting information can be downloaded at: https://www.mdpi.com/article/10.3390/ijms232415690/s1.
