# Supplementary material for: Metabolomics and Proteomics Characterizing Hepatic Reactions to Dietary Linseed Oil in Duck
Source: Int J Mol Sci. 2022 Dec 10;23(24):15690. doi: 10.3390/ijms232415690 (PMC9778787; doi:10.3390/ijms232415690)
Supplement: Supplementary file 1 [file ijms-23-15690-s001.zip › Supplement tables and figures.pdf]

Supplemental Tables and Figures

Supplemental Table S1 Differential metabolites in the positive ion mode

Metabolites	VIP	Fold change	*P* value
1-stearoyl-2-docosahexaenoyl-sn-glycero-3-phosphocholine	7.26	2.25	<0.0001
1-palmitoyl-2-docosahexaenoyl-sn-glycero-3-phosphocholine	15.12	2.58	<0.0001
Monolinolenin (9c,12c,15c)	3.00	2.82	<0.0001
1,2-dioleoyl-sn-glycero-3-phospho-l-serine	1.60	1.97	<0.0001
4-[5-[[4-[5-[acetyl(hydroxy)amino]pentylamino]-4-oxobutanoyl]-hydroxyamino]pentylamino]-4-oxobutanoic acid	1.68	1.54	0.0001
1,2-dioleoyl-sn-glycerol	1.28	1.77	0.0001
1-Stearoyl-sn-glycerol 3-phosphocholine	4.25	2.31	0.0002
2-docosahexaenoyl-1-palmitoyl-sn-glycero-3-phosphoethanolamine	1.92	2.73	0.0002
1-hexadecanoyl-2-octadecadienoyl-sn-glycero-3-phosphocholine	19.84	1.41	0.0003
2-oleoyl-1-palmitoyl-sn-glycero-3-phosphocholine	20.54	0.78	0.0003
Lpc 18:2	8.14	1.77	0.0005
1-palmitoyl-2-linoleoyl-sn-glycero-3-phosphocholine	12.62	1.77	0.0006
1-stearoyl-2-linoleoyl-sn-glycero-3-phosphoethanolamine	2.33	1.40	0.0010
Thioetheramide-PC	5.28	2.35	0.0011
1-hexadecyl-2-(8z,11z,14z-eicosatrienoyl)-sn-glycero-3-phosphocholine	1.76	2.26	0.0012
2-arachidonoylglycerol	1.42	0.59	0.0028
1,2-dilinoleoylglycerol	3.10	1.54	0.0032
Thymine	2.70	3.25	0.0035
Melezitose	7.03	0.36	0.0069
Erlose	1.10	0.34	0.0075
1-palmitoyl-2-linoleoyl-rac-glycerol	3.51	1.39	0.0083
1-Stearoyl-2-oleoyl-sn-glycerol 3-phosphocholine (SOPC)	1.06	0.86	0.0083
1-Stearoyl-2-arachidonoyl-sn-glycerol	1.30	0.39	0.0100
Stachyose	1.40	0.43	0.0107
Trigonelline	2.64	0.35	0.0108
PC(16:0/16:0)	7.63	2.12	0.0142
1,2-dioleoyl-sn-glycero-3-phosphatidylcholine	6.71	1.33	0.0164
1-oleoyl-2-myristoyl-sn-glycero-3-phosphocholine	2.42	0.67	0.0188
1-palmitoyl-2-hydroxy-sn-glycero-3-phosphoethanolamine	2.33	0.80	0.0194

Cytidine 5'-diphosphocholine	1.68	0.79	0.0202
.epsilon.-caprolactam	2.90	6.12	0.0267
Desisopropyldisopyramide	1.34	1.87	0.0286
2-linoleoyl-1-palmitoyl-sn-glycero-3-phosphoethanolamine	1.63	1.23	0.0337
Pro-leu	1.31	1.34	0.0361
Met-Met-Arg	1.21	0.54	0.0387
Tegaserod	2.63	1.59	0.0417
Pro-pro	1.83	0.41	0.0437
Hexanoyl-l-carnitine	1.64	0.52	0.0467
Linoleoylcarnitine	1.96	1.71	0.0478
1-octadecanoyl-2-octadecenoyl-sn-glycero-3-phosphocholine	11.67	0.84	0.0479

Supplemental Table S2 Differential metabolites in the negative ion mode

Metabolites	VIP	Fold change	*P* value
Pc(16:1e/17-hdohe)	3.35	2.28	<0.0001
Pc(18:1e/20-hdohe)	1.68	2.01	<0.0001
Ps 40:4	1.98	0.58	<0.0001
Linolenic acid	11.75	5.54	<0.0001
1-hexadecanoyl-2-(9z-octadecenoyl)-sn-glycero-3-phospho-(1'-myo-inositol)	3.97	3.26	<0.0001
Pc(18:1e/9-hode)	2.18	1.77	<0.0001
2-docosahexaenoyl-1-stearoyl-sn-glycero-3-phosphoserine	6.04	3.52	0.0001
Pe(16:1e/14-hdohe)	7.05	2.39	0.0002
Raspberry ketone	1.24	11.72	0.0002
1,2-distearoyl-sn-glycero-3-phospho-l-serine	3.77	2.21	0.0002
Ginsenoside rg5	4.43	7.92	0.0003
Cis-4,7,10,13,16,19-docosahexaenoic acid	8.26	2.75	0.0004
Ile-Pro	5.94	2.43	0.0004
1-palmitoyl-2-linoleoyl-sn-glycero-3-phospho-(1'-rac-glycerol)	1.70	0.77	0.0006
Pi 36:2	1.95	1.85	0.0008
Cis-7,10,13,16-docosatetraenoic acid	6.79	0.41	0.0009
4'-demethylpodophyllotoxin	1.13	0.33	0.0011
His-ser	6.91	3.94	0.0012

Pi 36:4	1.97	0.67	0.0016
Eicosapentaenoic Acid	1.03	2.21	0.0020
Hippuric acid	1.04	0.24	0.0028
Pyruvaldehyde	1.62	0.52	0.0040
Pe(16:1e/14,15-epete)	1.06	1.97	0.0042
NCGC00169011-01	5.52	7.32	0.0042
Linoleic acid	12.62	1.50	0.0053
Maltotriose	6.59	0.34	0.0055
Pg 40:5	1.31	0.60	0.0072
D-allose	15.48	0.52	0.0079
D-Maltose	2.53	0.39	0.0087
D-Sorbitol	2.08	0.51	0.0094
1-oleoyl-sn-glycero-3-phosphoethanolamine	1.14	0.75	0.0108
Gamma-Glu-Cys	16.32	0.17	0.0111
Dehydroascorbic acid (Oxidized vitamin C)	2.08	0.61	0.0114
Maltotetraose	1.48	0.45	0.0127
D-(+)-mannose	3.59	0.48	0.0134
Pc(16:0e/8-hepe)	3.27	0.71	0.0143
D-Tagatose	3.44	0.57	0.0160
Fahfa 36:4	1.13	2.21	0.0169
D-glucono-1,5-lactone	1.24	0.67	0.0178
Pg 36:3	18.56	1.39	0.0179
L-Glutamine	1.67	0.53	0.0184
Paxilline	3.26	3.58	0.0191
Phenylpyruvate	1.22	3.78	0.0234
Osmanthuside h	1.71	0.20	0.0282
1-palmitoyl-2-oleoyl-phosphatidylglycerol	1.93	0.78	0.0289
Xanthine	7.67	0.81	0.0325
L-pyroglutamic acid	2.01	0.60	0.0370
Pc(16:0e/5,6-eet)	1.33	0.79	0.0379
Cis,cis-muconic acid	4.70	1.17	0.0394
1-(9z,12z-octadecadienoyl)-2-hydroxy-sn-glycero-3-phosphoethanolamine	1.25	1.28	0.0414
Pe(16:0e/10-hdohe)	2.27	0.50	0.0437

Supplemental Table S3 Differential expression proteins between linseed oil group and the control group

Accession	Gene name	Protein description	HL/CL	*P* value
R0L721	Fibronectin type III and SPRY domain-containing protein 2 (Fragment)	Anapl_07374	7.08	0.0054
A0A493T552	Uridine-cytidine kinase	UCK1	6.21	0.0151
U3J3V0	Peptidyl-prolyl cis-trans isomerase	PIN1	5.49	0.0154
R0JLK7	Cytochrome P450 8B1 (Fragment)	Anapl_08712	4.33	0.0443
U3IEW1	Galectin	LGALS3	4.26	0.0208
R0L4X9	Membrane-associated progesterone receptor component 1	Anapl_02476	3.74	0.0166
U3J7S6	Uncharacterized protein		3.70	0.0196
A0A493T751	Cullin associated and neddylation dissociated 2 (putative)	CAND2	3.53	0.0237
R0KU23	Uncharacterized protein (Fragment)	Anapl_10169	3.49	0.0453
R0LCE7	Nucleolar complex protein 2 homolog (Fragment)	Anapl_09858	3.11	0.0288
R0LDI0	Gastric intrinsic factor	Anapl_15309	3.00	0.0003
R0LXV8	Sarcalumenin (Fragment)	Anapl_13158	2.76	0.0383
R0L7K1	Reticulocalbin-2 (Fragment)	Anapl_15847	2.76	0.0432
U3IKQ9	Ethanolamine-phosphate phospho-lyase	ETNPPL	2.70	0.0144
A0A493SUB3	Myosin IF	MYO1F	2.54	0.0125
R0L6N1	Histone H2A (Fragment)	Anapl_12366	2.42	0.0273
R0LGS0	Tax1-binding protein 1-like protein (Fragment)	Anapl_07940	2.39	0.0121
R0KAX8	Small monomeric GTPase (Fragment)	Anapl_15468	2.38	0.0413
A0A493U0U2	Kinesin family member 21A	KIF21A	2.35	0.0291
U3IDA2	WD repeat domain 3	WDR3	2.32	0.0105
R0LQ12	23 kDa cortical cytoskeleton-associated protein (Fragment)	Anapl_03819	2.30	0.0015
A0A493U208	Phosphatidate phosphatase	LPIN2	2.28	0.0135
A0A493TDB7	Interferon related developmental	IFRD1	2.26	0.0195

	regulator 1			
A0A493T3T7	Glycos_transf_1 domain-containing protein		2.24	0.0143
R0KUZ8	Putative aarF domain-containing protein kinase 5 (Fragment)	Anapl_18263	2.22	0.0086
R0JAZ4	ORM1-like protein 3 (Fragment)	Anapl_16259	2.19	0.0004
U3IAM4	Uncharacterized protein		2.13	0.0303
A0A493SWX0	SH3 domain-containing protein	SH3CP	2.12	0.0179
U3IJ50	Nuclear receptor coactivator	NCOA2	2.06	0.0053
R0LHG8	Vesicle transport through interaction with t-SNAREs-like protein 1A (Fragment)	Anapl_03516	2.03	0.0375
R0LPR2	U2-associated protein SR140 (Fragment)	Anapl_07670	2.02	0.0172
U3IH32	Kelch like family member 41	KLHL41	2.01	0.0433
R0JRJ5	Chloride channel protein (Fragment)	Anapl_03383	2.01	0.0125
A0A493TTK8	Uncharacterized protein		0.49	0.0024
R0KLG8	Ras-related protein Rab-5A (Fragment)	Anapl_15196	0.49	0.0040
R0KE13	Laminin subunit alpha-2 (Fragment)	Anapl_01843	0.48	0.0057
R0J7A6	Inositol-3-phosphate synthase (Fragment)	Anapl_18461	0.48	0.0403
R0LSM4	ES1 protein-like protein, mitochondrial (Fragment)	Anapl_12642	0.48	0.0246
U3IIN8	5-AMP-activated protein kinase catalytic subunit alpha-2	PRKAA2	0.47	0.0229
U3IE85	Peroxiredoxin-like 2 activated in M-CSF stimulated monocytes	PAMM	0.47	0.0449
A0A493SUY7	Arrestin beta 1	ARRB1	0.47	0.0340
U3J4Z7	Beta-mannosidase	MANBA	0.45	0.0480
A0A493SVI8	Uncharacterized protein		0.45	0.0134
R0JIG8	Actin-binding Rho-activating protein (Fragment)	Anapl_16253	0.45	0.0073
A0A493U0R0	S-adenosylmethionine synthase	MAT2A	0.44	0.0025
U3J9N5	Asparagine synthetase	ASNS	0.44	0.0215

	[glutamine-hydrolyzing]			
A0A493TZB0	Iso_dh domain-containing protein		0.42	0.0038
R0J6M7	Alcohol dehydrogenase 1 (Fragment)	Anapl_18753	0.41	0.0275
A0A493TUD7	SET and MYND domain containing 3	SMYD3	0.40	0.0178
U3IIW2	Fatty acid desaturase 2 (Fragment)	FADS2	0.39	0.0382
U3I1Q2	Complex I-23kD		0.39	0.0091
U3J4C8	Fatty acid binding protein 7	FABP7	0.38	0.0123
U3IBQ1	Phospholipid-transporting ATPase	ATP11C	0.38	0.0032
R0M1C6	Protein unc-45 homolog B (Fragment)	Anapl_01676	0.38	0.0151
A0A493SVN5	Actin binding LIM protein family member 3	ABLIM3	0.33	0.0253
R0K5A7	NADPH-dependent diflavin oxidoreductase 1 (Fragment)	Anapl_03059	0.30	0.0280
R0JU11	Centaurin-alpha-1 (Fragment)	Anapl_00457	0.26	0.0180
U3IW82	Dihydrolipoamide acetyltransferase component of pyruvate dehydrogenase complex	PDHX	0.25	0.0434
R0LE86	Intraflagellar transport protein 20-like protein (Fragment)	Anapl_18397	0.25	0.0190
U3IZG5	Myotilin	MYOT	0.24	0.0475
U3I4A4	Myopalladin	MYPN	0.21	0.0004
R0KVJ5	Oxoglutarate dehydrogenase (succinyl-transferring) (Fragment)	Anapl_18489	0.16	0.0308
U3IUW7	Calsequestrin	CASQ2	0.15	0.0487

Supplemental Table S4 Composition and nutrient level of the basic diet

Ingredient (%)	Content (%)	Nutrient level	Content (%)
Corn	48.12	AME（MJ/kg）	11.54
Soybean meal	24.72	CP（%）	17.88
Flour	18.02	EE（%）	5.88
Rice bran	5.10	CF（%）	3.66
Dicalcium phosphate	1.68	Ca（%）	0.91
Limestone	1.02	Total phosphorus（%）	0.77
Salt	0.31	Lys（%）	0.92
Premix[1]	1.02		
Total	100		

Note：AME: apparent matabolizable energy; CP: crude protein; EE: ether extract; CF: crude fiber; Lys: lysine. [1]Vitamin and mineral premixes supplied per kilogram diet: vitamin A, 9,000 IU; vitamin D_3, 3,000 IU; vitamin E, 79 mg; vitamin B_2, 8 mg; vitamin K_3, 2 mg; pantothenic acid, 3.2 mg; niacin, 11 mg; folic acid, 1.5 mg; biotin, 1 mg; Co, 1 mg; Mn, 49 mg; Cu, 6 mg; Zn, 60 mg; I, 2 mg; Se, 0.18 mg.

Supplemental Table S5 Fatty acid composition of the diets

Fatty acid (g/100g)	Control	High	Medium	Low	Duck oil	Linseed oil
TFA	5.00	4.94	4.72	4.87	70.32	68.13
SFA	1.55	1.48	1.37	1.29	19.94	7.17
MUFA	1.91	1.81	1.65	1.53	32.70	13.82
PUFA	1.54	1.65	1.70	2.05	17.69	47.15
ALA (C18:3n3)	0.08	0.21	0.36	0.68	0.67	35.21
Total n-6 PUFA	1.45	1.42	1.33	1.35	16.76	11.75
Total n-3 PUFA	0.08	0.22	0.36	0.69	0.82	35.37
n-6/n-3	17.37	6.51	3.64	1.97	20.37	0.33

Note: g/100 g, denominator represented fresh sample weight.TFA: total fatty acids, SFA: saturated fatty acid; MUFA: monounsaturated fatty acids; PUFA: polyunsaturated fatty acids; ALA: α-linolenic acid; n-6/n-3 PUFA: ratio of n-6 PUFA and n-3 PUFA.

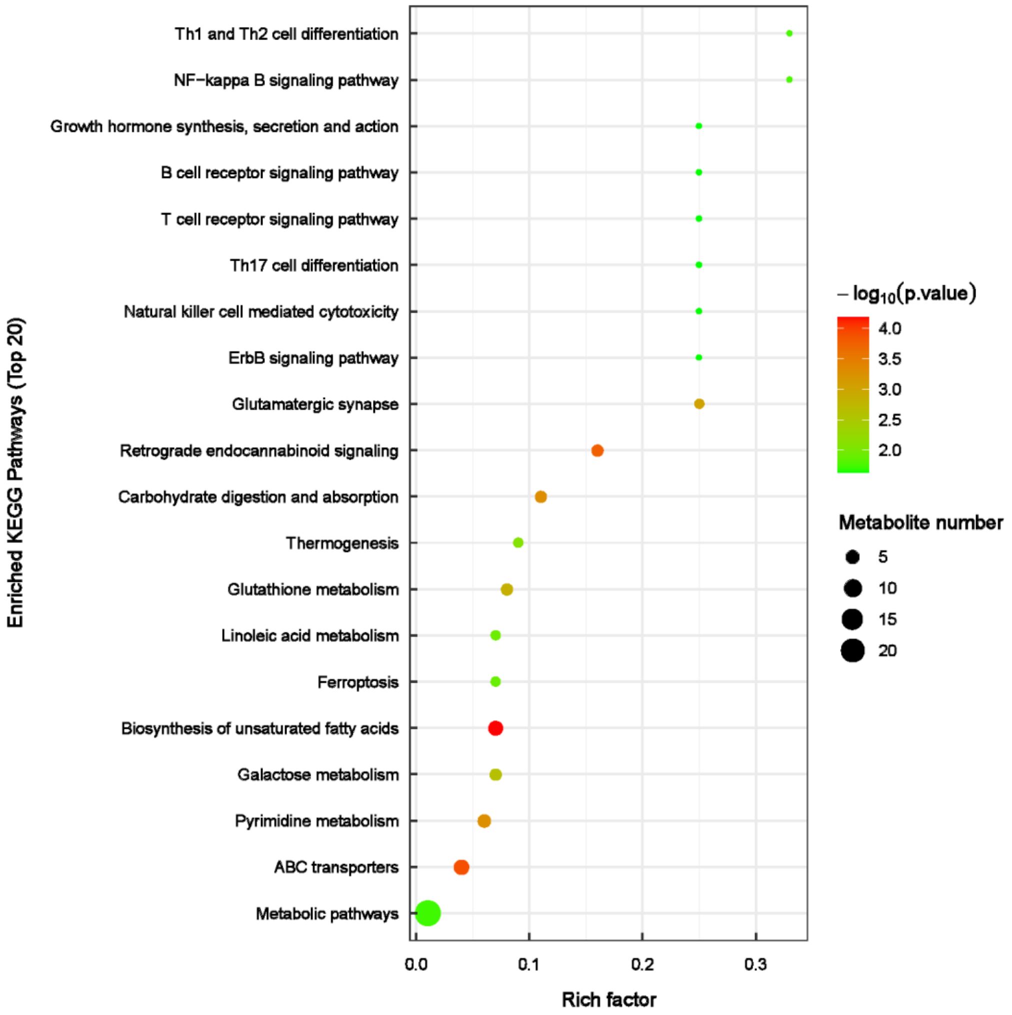

Supplemental Figure S1. KEGG pathway enrichment analysis of all differential metabolites.

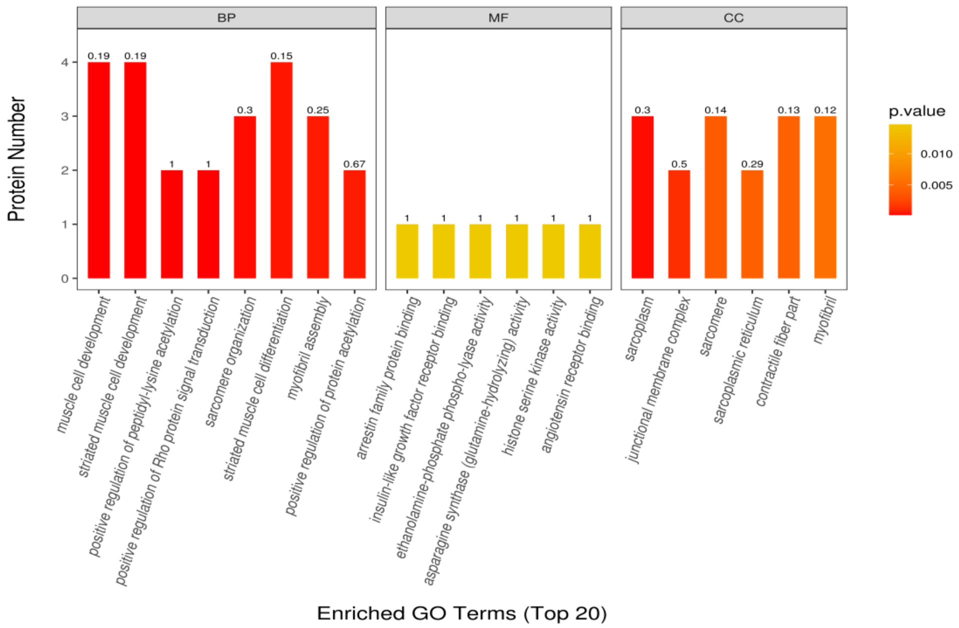

Supplemental Figure S2. GO annotation statistics of differential proteins.

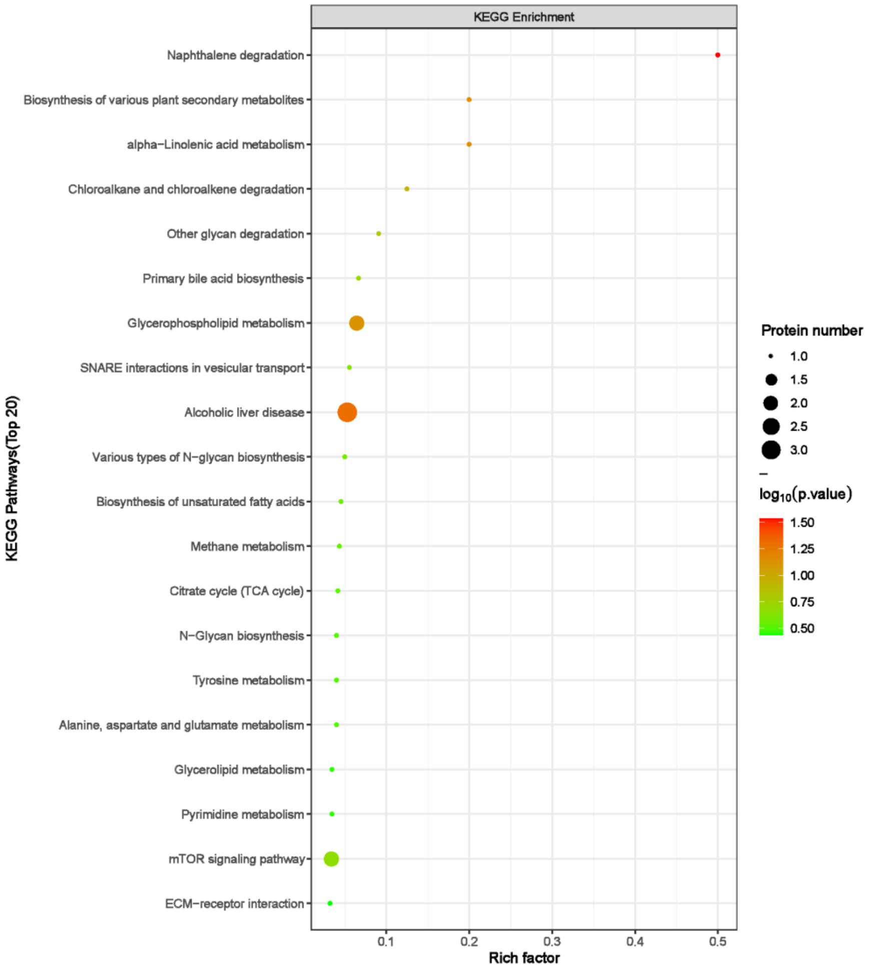

Supplemental Figure S3. KEGG pathway enrichment analysis diagram of differential proteins.

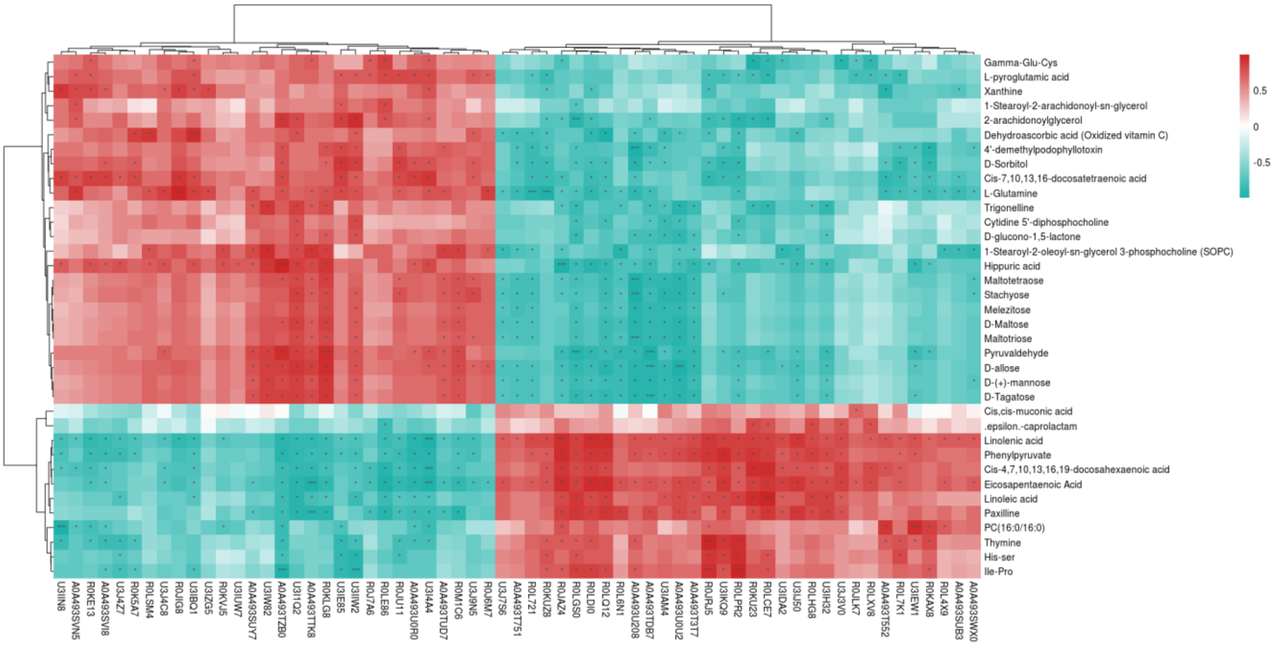

Supplemental Figure S4. Heat map of correlation analysis between the differential proteins and differential metabolites.

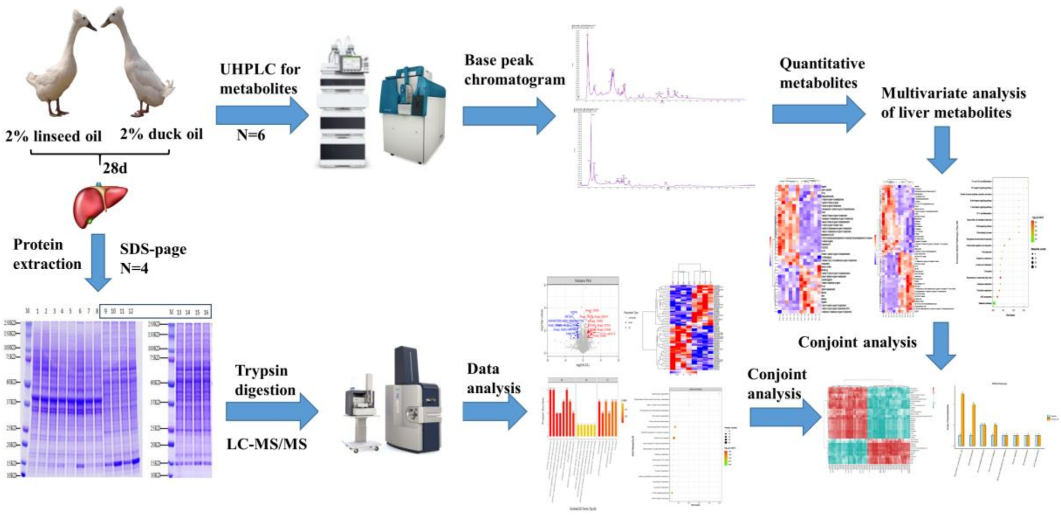

Supplemental Figure S5. Experimental design and workflow for the metabolomics and proteomics analysis of duck liver in the linseed oil group and the control group.
